# Machine Learning-Based Surrogate Model for Press Hardening Process of 22MnB5 Sheet Steel Simulation in Industry 4.0

**DOI:** 10.3390/ma15103647

**Published:** 2022-05-20

**Authors:** Albert Abio, Francesc Bonada, Jaume Pujante, Marc Grané, Nuria Nievas, Danillo Lange, Oriol Pujol

**Affiliations:** 1 Eurecat, Centre Tecnològic de Catalunya, 08005 Barcelona, Spain; francesc.bonada@eurecat.org (F.B.); jaume.pujante@eurecat.org (J.P.); marc.grane@eurecat.org (M.G.); nuria.nievas@eurecat.org (N.N.); danillo.lange@eurecat.org (D.L.); 2 Department de Matemàtiques i Informàtica, Universitat de Barcelona, 08007 Barcelona, Spain; oriol_pujol@ub.edu

**Keywords:** surrogate modeling, intelligent manufacturing, machine learning, press hardening, simulations, sheet metal forming, Industry 4.0

## Abstract

The digitalization of manufacturing processes offers great potential in quality control, traceability, and the planning and setup of production. In this regard, process simulation is a well-known technology and a key step in the design of manufacturing processes. However, process simulations are computationally and time-expensive, typically beyond the manufacturing-cycle time, severely limiting their usefulness in real-time process control. Machine Learning-based surrogate models can overcome these drawbacks, and offer the possibility to achieve a soft real-time response, which can be potentially developed into full close-loop manufacturing systems, at a computational cost that can be realistically implemented in an industrial setting. This paper explores the novel concept of using a surrogate model to analyze the case of the press hardening of a steel sheet of 22MnB5. This hot sheet metal forming process involves a crucial heat treatment step, directly related to the final part quality. Given its common use in high-responsibility automobile parts, this process is an interesting candidate for digitalization in order to ensure production quality and traceability. A comparison of different data and model training strategies is presented. Finite element simulations for a transient heat transfer analysis are performed with ABAQUS software and they are used for the training data generation to effectively implement a ML-based surrogate model capable of predicting key process outputs for entire batch productions. The resulting final surrogate predicts the behavior and evolution of the most important temperature variables of the process in a wide range of scenarios, with a mean absolute error around 3 °C, but reducing the time four orders of magnitude with respect to the simulations. Moreover, the methodology presented is not only relevant for manufacturing purposes, but can be a technology enabler for advanced systems, such as digital twins and autonomous process control.

## 1. Introduction

The press hardening of a steel sheet, also known as hot stamping, is a thermomechanical forming process in which sheet steel in austenitized conditions (typically in a 890–950 °C range) is transferred to a set of cooled dies to be formed and quenched in a single step, resulting in a fully martensitic structure with up to 1500 MPa tensile strength [1,2]. This technology offers interesting compromises, allowing to obtain components with very high mechanical properties and great shape complexity, in a cost-effective manner, and all but bypassing the issues of spring-back and the lack of formability typical in high-performance materials.

In the last two decades, press hardening has become the reference technology in lightweight safety cage body-in-white applications, with ultra-high-strength steels (UHSS) being extensively used in safety-related components, such as A- and B-pillars or frame dash panels. The most common UHSS for these purposes are 22MnB5 and, more recently, 37MnB5 [3]. Moreover, by its very definition, press hardening involves a heat treatment, meaning that both geometry and mechanical properties are generated on the press floor. This has led to consistent interest in ensuring part quality and traceability, with the constant need to improve experimental testing methods [4]. Additionally, this interest has brought press hardening to be a prime objective for Industry 4.0 concepts.

The Fourth Industrial Revolution has transformed the manufacturing industry, leading to a digitalization process that has driven the establishment of a complete connection between manufacturing elements and has enabled the effective exploitation of manufacturing processes data [5]. Thereby, the Industry 4.0 paradigm includes the integration of sensors, computing platforms, communication technologies, data-intensive modeling, control systems, and simulation and predictive tools to manage processes in real-time [6].

Industry 4.0 has also been the driver for new promising technologies. The most popular being the digital twin [7], which consists of a mirror version of the real system in a digital environment. The key characteristic of the digital twin is the connection between the real and the digital version of the system, which allows the data exchange in real time [8].

In addition, these concepts can be further reinforced by the application of artificial intelligence (AI) and machine learning (ML) in manufacturing [9,10]. For instance, supervised learning has been shown to be an effective tool to handle the production data, providing additional information from the human expertise and the quality control protocols [11,12]. Moreover, it is used for quality [13] and process monitoring [14]. On the other hand, unsupervised learning has the relevant characteristic of being capable of identifying patterns to handle the important quantity of unlabeled data that is generated in manufacturing. Unsupervised learning has achieved great results in anomaly detection [15], fault and quality prediction [16], and predictive maintenance [17], among others. Furthermore, more sophisticated approaches have been explored in the literature, such as generative adversarial networks (GANs) or the hybrid combination between different ML techniques. Some of the applications of GANs have been the anomaly detection [18] and the augmentation of small experimental datasets with artificial data to feed ML algorithms in prognostics and health management [19]. Meanwhile, the combination of decision trees and artificial neural networks (ANNs) has been used in lifetime prediction [20]. Nevertheless, the most novel approach is reinforcement learning, which, despite being at an early stage, has shown great potential in industrial manufacturing applications [21]. The reason is that it is able to provide goal-directed learning oriented to decision making. Its applications are still limited, mainly focused on planning, scheduling optimization, production maintenance, and quality control [22].

However, as previously discussed for digital twins, the generation and accessibility of process data and its posterior treatment are crucial for the mentioned ML techniques to work properly [23]. The possibility of performing real experiments of the processes to acquire data is often unfeasible, since they are costly and imply the waste of raw materials. In this context, manufacturing simulations, mainly finite element modeling, are the main source of data and knowledge without perturbing the real manufacturing system [24]. Simulations enable the exploration of new scenarios and configurations, as well as the modification of the experimental conditions.

Finite element modeling is a mature technology, commonly used in the manufacturing industry. In the particular case of press hardening, finite element modeling has been in widespread use since the early 2000s [25], and has steadily evolved to capture complex aspects such as thermal and mechanical interactions [25], plastic flows at different temperatures [26], and eventually phase transformations and the behavior of different microconstituents [27]. Currently, the simulation of press hardening can be readily performed with commercial software [28] with industrially relevant results. However, despite their obvious advantage in front of real-world experiments, simulations are still a complex and time-intensive tool that cannot be realistically run in real time or used to generate very large libraries of data. This supposes a limitation in the design of technologies such as digital twins, which require a huge amount of simulations for the training of the data analysis tools. Besides, the simulations in the digital environment must provide a rapid response in order to predict the optimal operational configuration of the system [29]. In the same way, in fields such as autonomous process control, the development of reinforcement learning agents is starting to be relevant [30], which demands a high volume of data that is hardly workable in terms of time and resources with the simulation methods.

To overcome the mentioned limitations of the manufacturing simulations, it is possible to combine simulations and ML in a hybrid approach to build a highly efficient model which acts as a surrogate of the simulations [31]. These surrogate models, or advanced response surfaces, are meta-models that aim to describe a system or a process in a simpler but representative way. The aim of surrogate models is to give an approximation of a function that relates input variables with output target variables to offer a faster response than the one that a complete simulation model provides. The surrogate models can be generated using real-world data or simulation data [32,33,34]. Despite the simplicity of the model, the response is helpful for the understanding and the optimization of the process. Usually, in manufacturing, with a few relevant variables, it is possible to evaluate the performance of the manufacturing system through the key performance indicators (KPIs).

KPIs are metrics that quantify the performance of a manufacturing process over time in manufacturing [35]. Some of the most important KPIs are overall equipment effectiveness (OEE) [36], scrap rate [37], and cycle time [38], among others. OEE identifies the percentage of truly productive times, speeds, and qualities. It is an indicator used to determine how well equipment is used in batch production and it is related to losses that can impede the equipment efficiency. Differently, scrap rate measures the defective products that are useless and cannot be restored with respect to the total of the batch production. Finally, cycle time is the amount of time that a process lasts in fabricating a product.

The possibility of implementing ML to process control in press hardening has been proposed in the literature and specialized fora, with different approaches being considered [39,40,41]. The basic common ground tends to heavily lean into monitoring process temperature at different points, thus ensuring that the heat treatment and final part properties are controlled. Differently, this work presents a novel approach to built a data-driven surrogate model of the press hardening process of the UHSS 22MnB5. The aim is to demonstrate the model validity to predict the performance of a simplified press hardening process reproduced in a finite element modeling environment, offering a much faster response than in the simulations. The model analyses a problem where an austenitized piece of sheet steel is quenched using water-cooled steel dies, reproducing the experimental setup of a real industrial plant described in reference [42]. The surrogate model focuses on the prediction of the most relevant target variables for the process, which determine the quality of the obtained sheet and the state of the press hardening die.

The model consists of a supervised ML algorithm, which establishes relationships between the input variables of the process and the target variables. The training of the model is performed with a series of finite element simulations performed inside a pre-defined parameter space. Concretely, two training methodologies are proposed: one built with simulations inside the typical operation framework and the other covering non-standard cases. Another purpose of the work is to propose an efficient method to train the surrogate model in order to achieve the maximum generalization capability in the validation process. The validation scenarios are defined by adjusting the parameters to the facilities of the real industrial plants, but also explore new operation possibilities towards dynamic process optimization. The results show that the surrogate model trained with non-standard cases is more suitable for the prediction in all the evaluated scenarios and it can be optimized with the objective to reduce the number of FE simulations required in the training phase. The key advantages of the surrogate modeling of the press hardening process is that it provides a soft real-time response of the target variables of the process and enables the creation of time- and cost-efficient virtual environments for knowledge collection, overcoming the time and computational limitations of traditional FE simulations.

## 2. Materials and Methods

### 2.1. Press Hardening Process

Press hardening, also known as hot stamping, is a hot sheet metal forming process, which consists of the austenitization of a sheet steel at a temperature between 900 and 950 °C inside a furnace. Afterwards, the sheet is transferred into a set of cooled dies where it is formed while its temperature is inside the 650–850 °C range. Then, the dies are kept close and pressure is applied for a short period of time. During this step, the cooled dies quench the formed component at a cooling rate between 50 and 100 °C/s to a temperature of 100–250 °C, ensuring full martensitic microstructure [1]. The finished component is then extracted from the die. The total cycle time includes transfer, forming, and quenching typically.

In this work, a simplified hot stamping process is analyzed by finite element modeling (FEM) using the ABAQUS software [43], based on the experimental layout discussed in reference [42]. The reproduced setup consists of a flat water-cooled die made out of steel DIN 1.2344 (roughly corresponding to AISI H13) tempered at 48 ± 1 HRC, with water channels 10 mm in diameter and located at 20 mm depth from the surface, with a separation of 50 mm between centers. On these tools, an austenitized 22MnB5 sheet 1.7 mm in thickness is introduced, and the dies are closed, resulting in the component being quenched. The chemical composition of the two materials is presented in Table 1.

**Figure 1:**
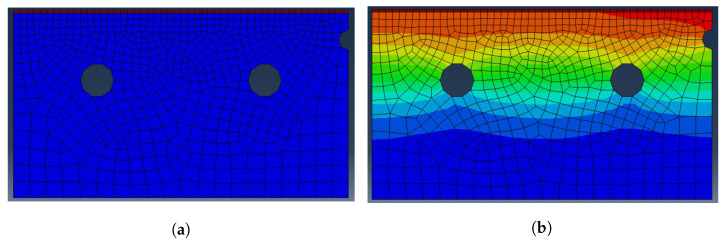
Representation of the mesh temperature profile in ABAQUS. (**a**) Initial state. The hot sheet is displayed in red and the cold die in blue. (**b**) State after a forming phase. The temperature profile is difficult to differentiate between the sheet and the die.

Simulation 2D models have been created with a focus on economy of calculation, as the main aim of the work is to generate a very large amount of simulations to demonstrate the surrogate models. Transient heat transfer analysis is realized with a model meshed with four-node linear quadrilateral elements and using a slightly higher mesh density in contact boundary regions. Quadrilateral-shaped elements have been used instead of triangular to reduce the number of nodes involved in the model, consequently reducing the computational cost. A total of 783 elements and 995 nodes has been used to represent a transversal cut of the sheet and the die during the process, using the system symmetry to further simplify geometry, as shown in Figure 1. Plastic deformation and phase changes are not considered, reducing the scenario to a heat transfer problem representative of a local analysis of quenching of a press-hardened component. For the same reason, strategies to increase precision of results such as local mesh refinement are not employed, instead performing the whole batch of simulations using the basic-defined mesh.

For this model, the main material properties simulated have been density, estimated at 7800 kg/m^3^ for both steels and heat conductivity, where values of 23 W/m·K for 22MnB5 steel and 27 W/m·K for 1.2344 have been used in accordance to references [26,44]. Thermal contact conductance between dies and sheet metal has been set at 3000 W/m^2^·K, as used in reference [45]. A boundary film condition has been applied on inner surfaces of die cooling channels. A 12,000 W/m^2^·K heat transfer coefficient and 25 °C of sink temperature were used regarding the turbulent flow of a water-cooled system, created with drilled channels.

Using this model, a series of heat transfer transient simulations are run sequentially. Then, the die temperature changes along the cycles. The initial die temperature is set to to 25 °C; from that point, each cycle uses the temperature distribution on the die resulting from the previous simulation. In this manner, die heating is reproduced in the simulation model as it is observed in the physical system. On each cycle, a new sheet is considered, with an initial temperature of 800 °C, a reasonable estimation of an industrial press hardening process. A cycle simulation comprises two phases:

1. Forming phase: It represents the stage when the die is closed and there is a heat transfer between the hot sheet and the cold die: this phase is governed by the forming time;
2. Cooling phase: The sheet has already been extracted. It includes the recovery of the die after the forming phase and the transfer of the next metal sheet in the die. This phase is governed by the cooling time.

**Figure 2:**
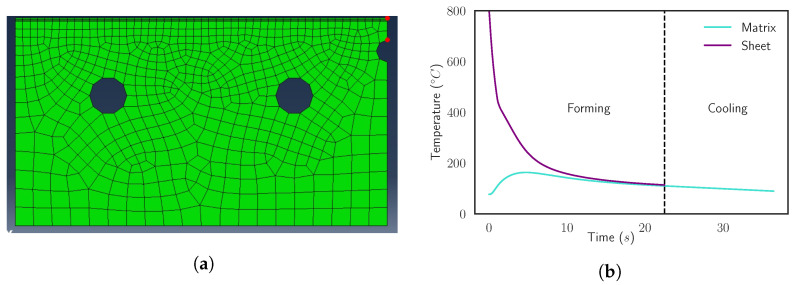
(**a**) Location of the reference nodes in the mesh. The upper red point is the sheet reference node (S) and the lower red point is the die reference node (D). (**b**) Evolution of the reference points’ temperatures during a complete press hardening simulation cycle.

Despite Figure 1, where the distribution of all the node temperatures is displayed, two nodes are taken as a reference for the sheet and die temperatures during the process. In Figure 2a, the location of these nodes in the mesh is indicated and it corresponds to the position of real sensors. As a result, the simulations allow to control the temperature of the sheet and the die of the press hardening process from a similar point of view to the industrial plant. The behavior of the reference nodes in an example simulation cycle is shown in Figure 2b.

The press hardening simulations demand some input values that are restricted to the conditions of the real industrial plant. The most relevant input variables are the following:

- Initial distribution of the sheet temperature. We focus on the temperature at the reference node $T_{ini}^{S}$. It is the temperature of the sheet at the start of the forming phase. It is assumed to be 800 °C in this study;
- Initial distribution of the die temperature. We focus on the temperature at the reference node $T_{ini}^{D}$. It is the temperature of the die at the start of the forming phase. It is assumed to be 25 °C in the initial cycle, but it keeps changing when sequential cycles are simulated. It represents the actual state of the press hardening system at the start of the cycle;
- Forming time $t_{form}$. It refers to the duration of the forming phase. To ensure the quality of the sheet, the forming time has a minimum value of $t_{form}=10$ s in the industrial plant;
- Cooling time $t_{cool}$. It refers to the duration of the cooling phase. The transfer cannot be immediate and a cooling of the die is required. Then, $t_{cool}$ ranges from [10to20] s in the current industrial plant;
- Cycle time $t_{cycle}$. It is the total duration of a simulation cycle. $t_{cycle}=t_{form}+t_{cool}$. The real plant restricts this variable in the interval of [30,40] s. In this plant, the sheets go through the furnace in a belt and cycle time depends on the furnace providing the next hot sheet. In more advanced industrial plants, the cycle time range is wider, because they use several furnaces at the desired temperature, and the hot sheet is available anytime.

At the end of a simulation cycle, there are three output variables which provide crucial information about the realized press hardening cycle. These target variables determine the state of the system, and the quality and the good performance of the press hardening process:

- Final distribution of the die temperature. We focus on the temperature at the reference node $T_{fin}^{D}$. It is the temperature of the die at the end of the cycle after the forming and cooling phases. In a sequential simulation of cycles it keeps evolving and it is the value for the $T_{ini}^{D}$ of the next cycle. Therefore, it represents the actual state of the press hardening system at the end of the cycle;
- Final distribution of the sheet temperature. We focus on the temperature at the reference node $T_{fin}^{S}$. It is the temperature of the sheet at the end of the forming phase when the sheet is extracted. This variable controls the quality of the final product. If it exceeds a threshold temperature, the sheet has not been able to acquire the martensitic microstructure due to a slow cooling;
- Distribution of the maximum die temperature. We focus on the temperature at the reference node $T_{max}^{D}$. It is the maximum temperature achieved in a cycle. This variable makes sure that die capacities are not exceeded and ensures that it is able to support the process.

In general, in industrial plants, the execution of only a single cycle of press hardening is not the usual way of operation. The demand requires the production of batches of more than one product, which implies carrying out several cycles. In the current work, 50 sequential cycles are equivalent to a batch. Therefore, to build the surrogate model, we generate batch simulations of 50 press hardening cycles to mimic a possible real-demand case.

Although the industrial plant characteristics limit the cycle time to a range of values, the surrogate model aims to generate an environment to look for the optimization of this feature and the product quality in the batches. The modification of forming and cooling times could lead to a change in the cycle time, but also could imply the manufacturing of a defective sheet. Hence, there is a trade-off between the reduction of the total cycle time in the batches and the final quality of the metal sheets. Since the transference of the sheet from the furnace into the die is usually performed by an automated system, in the study we focus on the creation of a surrogate model able to reproduce scenarios where the cooling time is set constant, according to the possibilities of real industrial plants. Then, the forming time is modified, affecting the total cycle time. The forming time can be changed in a real plant, controlling the duration of the die closure. In Figure 2b, it is shown how a change of the forming time influences in the $T_{fin}^{S}$ and the $T_{fin}^{D}$. Summarizing, in this work, the surrogate model opens the door to explore a possible real-operation scenario where we try to optimize the time and the quality of the metal forming process, modifying the forming time of the cycles while keeping the cooling time constant.

### 2.2. Surrogate Modeling of the Process

This study proposes a methodology for the development of a data-driven surrogate model, consisting of a supervised machine learning regression algorithm. The surrogate model is built using simulations from the high-fidelity FE simulations described in Section 2.1. The simulations consist of different batches of 50 cycles. Ideally, the surrogate model should be able to reproduce batches in the whole region of the parameter space. To achieve this generalization capability, we have generated two candidate training sets under different simulation conditions in order to evaluate which provides the most general surrogate model.

**Figure 3:**
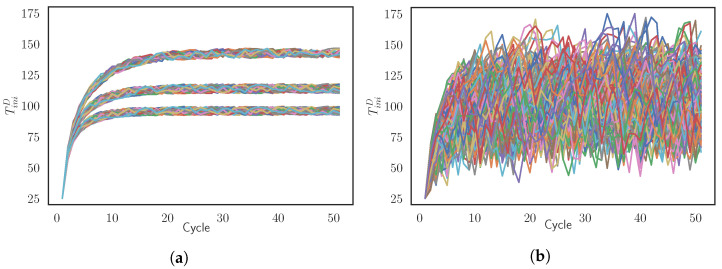
Representation of the evolution of $T_{ini}^{D}$ for the simulated batches in the training sets (**a**) A and (**b**) B.

- **Training Set A:** The parameters of the simulations agree with real-operation conditions. Each cycle lasts randomly within $t_{cycle}=\left[30,40\right]$ s, with a discretization of the interval each 0.5 s. The cooling time is set constant along the batches according to the scenario that we want to reproduce in this study. Therefore, the training set contains 90 batches of $t_{cool}$ = 10 s, 15 s, and 20 s, respectively. The forming time oscillates depending on the random value of $t_{cycle}$, fulfilling the restriction of $t_{cycle}=t_{form}+t_{cool}$. This training set A it is used to feed the Surrogate Model A (SModA). Figure 3a shows the evolution of $T_{ini}^{D}$ of the simulations of the Training Set A;
- **Training Set B:** The simulations do not correspond to normal operation conditions. In this case, the cycle time also has a random value within $t_{cycle}=\left[30,40\right]$ s, discretized each 0.5 s. Moreover, the cooling time has a random values for each cycle ranged in the interval $t_{cool}=\left[0,40\right]$ s, with a discretization of 0.5 s. The forming time is the result of the condition $t_{cycle}=t_{form}+t_{cool}$. This dataset is formed by 270 batches and it is used to train the Surrogate Model B (SModB). Figure 3b shows the evolution of $T_{ini}^{D}$ of the simulations of the Training Set B.

Both surrogate models are based on supervised regression ML algorithms. In Section 2.1, we have determined the most important input variables of the process and the most relevant output variables. To mimic the simulation, the surrogate models have the same inputs and outputs. The inputs are $T_{ini}^{S}$, $T_{ini}^{D}$, $t_{form}$, and $t_{cool}$ and the target variables are $T_{fin}^{D}$, $T_{fin}^{S}$, and $T_{max}^{D}$. However, there exist a slight difference between the SModA and SModB. The SModA also adds in its inputs a temporal window with the 3 previous values of the variables $T_{ini}^{D}$, because, in Figure 3a, the simulations are shown to have a temporal dependence. Several supervised regression algorithms are implemented using the Scikit-learn [46] and the XGBoost [47] Python libraries. Concretely, 4 candidate algorithm are explored: K-nearest neighbors (KNN) [48], based on Euclidean distance as similarity metric; support vector regressor (SVR) [49], which works with hyperplanes in the dimensional space defined by the input data; and extreme gradient boosting (XGBoost) [47] and random forest (RF) [50], which are ensemble techniques. These candidate algorithms cover some of the most-used types of supervised ML algorithms. A 5-fold cross-validation [51] is applied to check the performance of the algorithms in both training sets in order to determine the best one.

As observed, there is a huge difference when comparing Figure 3a, whose simulations are performed under standard operation conditions, with Figure 3b. In Training Set B, the simulations do not cover standard cases, but they explore a wider region of possible states of the system. The surrogate models are validated with simulations that are analogous rather than the ones forming Training Set A, with distinct parameter values according to the facilities of our industrial plant. The FE simulations act as our ground truth. Finally, we identify which is the better option to built a surrogate model capable to generalize unseen simulation data.

## 3. Results and Discussion

In this section, we evaluate the surrogate models created with the different methodologies proposed in previous sections, presenting the results of the accuracy of the models in the prediction of the target variables. In addition, we try to optimize the simulations that are required to feed the surrogate model, to achieve a reduction of the simulation time without having a significant impact in the quality of the models.

As emphasized in Section 2.1, the state of the system after a press hardening process is determined by the final die temperature, $T_{fin}^{D}$. Consequently, the evolution of this variable governs the evolution of the whole system. Since it is used as input for the surrogate model, a poor prediction of this variable affects the next cycles prediction of the other target variables, which establish the metal sheet quality and the smooth operation of the system. For that reason, we give numerical results for all the target variables, but, as an insight, we display figures only for the temperature of the die. The figures for the rest of target variables are presented in the Appendix A.

### 3.1. Baseline Prediction Results

Both surrogate models are based on regression algorithms that predict the state of the system and the most relevant process variables after a complete cycle of press hardening. To choose an algorithm we implement the technique of five-fold cross-validation [51] to check the performance of various candidate algorithms on both training sets. The metric for algorithm evaluation used in this work is the mean absolute error [52]:

(1) $$\mathrm{MAE}=\frac{\sum_{i=1}^{N}\left|x_{i}-{\hat{x}}_{i}\right|}{N}$$

where $x_{i}$ are the actual values, ${\hat{x}}_{i}$ are the predicted values, and *N* is the number of samples.

As shown in Table 2, the best algorithms are able to obtain very good results for all the target variables. The values of MAE are less than 0.5 for Training Set A and less than 2 for Training Set B, without a relevant SD. Therefore, the XGBoost algorithm acts as the basis of the surrogate model during the rest of the work. The results of the five-fold CV of this regression algorithm in both training sets represent a baseline for the creation of a surrogate model. The next step is the evaluation of the surrogate model in validation sets corresponding to the real-plant framework, with the objective of generating a general surrogate model capable to predict the target variables in the all the regions of the parameter space.

### 3.2. Exploration of Validation Scenarios

The validation of the surrogate models is performed according to different situations that may be encountered in a real industrial plant. Then, we have several validation scenarios to compare the performance of the two surrogate models and determine which is the best model able to generalize in various regions within the range of the parameter space. It must be noticed that some of the validation scenarios are built using the same simulation conditions, rather than Training Set A or B. In these cases, it is unfair to compare the SModA performing in a validation set with the conditions of Training Set A, and the same happens for SModB and Training Set B conditions. However, this issue has been introduced with the purpose of validating the SModA in the training conditions of the SModB and vice versa. In this way, the generalization capability of a surrogate model in the prediction of unseen scenarios compared with an unfair prediction is remarked upon.

#### 3.2.1. Single-Cycle Prediction

The final metal sheet quality and the die state are the most significant features after a press hardening process. The resulting temperatures of the sheet $T_{fin}^{S}$ and the die $T_{fin}^{D}$ provide this information. Additionally, the control of the maximum temperature of the die $T_{max}^{D}$ during the process ensures that the die has not exceeded its operational window. The simulation of a single cycle of a forming process provides these target variables as outputs. Hence, we expect the surrogate model to accurately predict the same target variables after a process without the need of the simulation, under different input conditions. Each of the three validation sets consists of 500 samples, which gives a total ratio of ∼1:8 with respect to the training sets.

Validation Scenario 1: $t_{cool}$ = 10 s, 15 s and 20 s.

The validation set is formed by 500 randomly input samples obtained from simulations under the conditions of Training Set A. We do not include samples from the first three cycles in the set, since the surrogate model fed with Training Set A requires information about the three previous cycles to work. In this scenario, the initial die temperature varies between 80 and 150 °C, the cooling time have values of $t_{cool}$ = 10 s, 15 s, and 20 s, and the forming times have a random value with the restriction of the cycle time $t_{cycle}=t_{form}+t_{cool}=\left[30,40\right]$ s.

**Figure 4:**
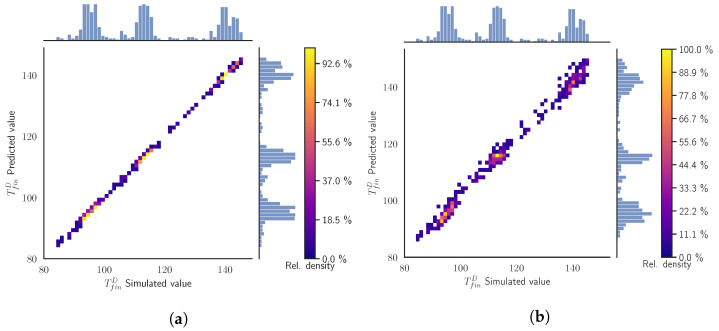
Validation Scenario 1: Predicted values as function of the simulated output values of the $T_{fin}^{D}$. The histograms and the color map represent the relative counts as function of the temperature. (**a**) SModA and (**b**) SModB.

Figure 4 presents the surrogate models’ prediction values for the $T_{fin}^{D}$ versus the simulated values obtained from the simulation using the same inputs. The axis of the plot are divided into 50 bins to build a histogram of the distribution of $T_{fin}^{D}$ for both the predicted and simulated values, which act as our ground truth. This divides the space in the cells that are colored according the relative density of the samples compared to the cell with the maximum number of samples. For instance, 100% of relative density in a cell means that there are the same number of samples, rather than in the cell with the maximum number of samples. The figure additionally shows the empirical distributions of the simulated values (at the top of the figure) and the predicted ones (at the right side of the figure). In the ideal case, both distributions should be the same.

In Figure 4a, we appreciate a narrow line following the diagonal of the plot, implying an almost perfect prediction from the SModA. Observing the empirical distributions, we see that the zones with more density correspond to the values of the $T_{fin}^{D}$ in the stationary regime for the cases $t_{cool}$ = 10 s, 15 s, and 20 s, as can be seen in Figure 3a. The high prediction capability of the SModA in this validation scenario was expected, as training and validation sets share the same $t_{cool}$ values. On the other hand, despite the randomness in its training, the SModB is able to approach the diagonal line and also correctly captures the zones with more density, as it is shown in Figure 4b. Nevertheless, the dispersion of the points indicates that the predictive power is lower than in the other case.

These features are repeated for the other target variables $T_{fin}^{S}$ and $T_{max}^{D}$. The respective figures of these variables are displayed in the Appendix A. The results are condensed in Table 3, where it is evidenced how the SModA outperforms the SModB in this particular validation case for all the target variables, although the SModB does not show very high values of the MAE. An error of about 2 °C is not unfeasible in experimental conditions, and can be often present due to systematic errors or calibration issues.

Validation Scenario 2: $t_{cool}=$ Intermediate values.

In this case, the validation set contains 500 randomly selected input points from simulations with intermediate values of $t_{cool}$, rather than the ones in Training Set A. The same as before, we do not add points from the first three cycles in the set, taking into account the limitation of the surrogate model trained on Training Set A. Then, the initial die temperature ranges between 85 and 145 °C, the forming times have values of $t_{cool}$ = 11 s, 12 s, 13 s, 14 s, 16 s, 17 s, 18 s, and 19 s, and the forming time has a random value with the restriction of the cycle time $t_{cycle}=t_{form}+t_{cool}=\left[30,40\right]$ s.

**Figure 5:**
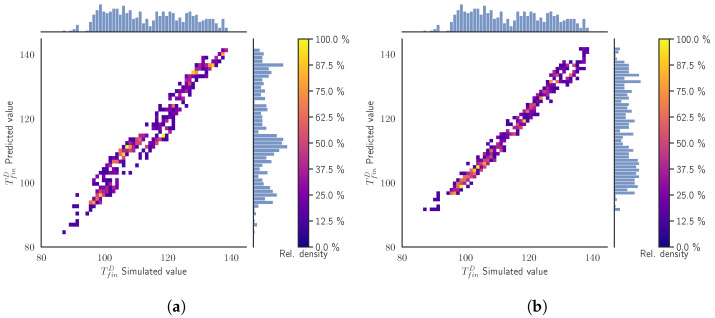
Validation Scenario 2: Predicted values as function of the simulated output values of the $T_{fin}^{D}$ under. The histograms and the color map represent the relative counts as function of the temperature. (**a**) SModA and (**b**) SModB.

In the current validation scenario, the SModA does not perform as well as in the previous case. The intermediate values of $t_{cool}$ force the model to make an interpolation. In Figure 5a, the points are distributed around the diagonal, although they form a line with a significant width, meaning more prediction error and SD. The SModB presents a narrower line around the diagonal, as it can be observed in Figure 5b. We notice that the intermediate values of $t_{cool}$ cause a more uniform density distribution along the range of temperatures.

Table 4 shows the commented results of the SModB for the variable $T_{fin}^{D}$. It must be noted that for the rest of the target variables, SModA has a lower MAE. Nonetheless, also taking into account Figure 5, we consider that SModB is better in the prediction of the $T_{fin}^{D}$ of a next cycle than SModA in this parameter interpolation case, but observing the SD of both models, we observe that the overlap in the results makes it difficult to establish a clear option.

Validation Scenario 3: $t_{cool}=$ Random.

The validation set consists of 500 randomly sampled input points obtained from simulations under the conditions of Training Set B. Again, for the same reason as before, the first three cycles are not included in the set. In this case, the initial die temperature of the samples ranges between 50 and 165 °C, while the forming and cooling times range between $t_{form}\;\mathrm{and}\;t_{cool}=\left[0,40\right]$ s, with the restriction of the cycle time $t_{cycle}=t_{form}+t_{cool}=\left[30,40\right]$ s.

**Figure 6:**
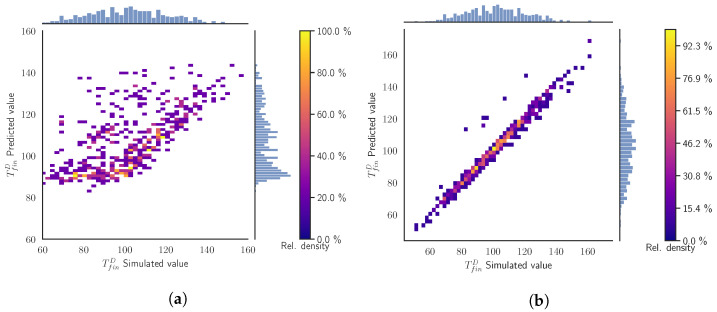
Validation Scenario 3: Predicted values as function of the simulated output values of the $T_{fin}^{D}$. The histograms and the color map represent the relative counts as function of the temperature. (**a**) SModA and (**b**) SModB.

In Figure 6a, we see that in a random scenario the SModA performs poorly due to its lack of information about some regions of the parameter space. We observe a high dispersion of the points and the diagonal has nearly disappeared. Otherwise, as expected, Figure 6b shows that the SModB maintains its good performance. With a few exceptions, almost all the points are condensed around the diagonal, meaning that the predictions are very close to the simulation values. The training under random conditions results in a high adaptability to any value of the input variables. Checking the other target variables in Table 5, we confirm that SModB outperforms SModA in this more general scenario.

Summarizing, we identify that the SModA is able to carry out good predictions of the next cycle target variables in the exactly same training regimes, specifically, cases when $t_{cool}=\left[10,20\right]$ s. However, the SModB achieves reasonably good performances in all the validation scenarios, showing a constant and controlled behavior.

#### 3.2.2. Batch Prediction

Usually, in industrial manufacturing, the demand requires several press hardening processes to obtain a batch consisting of a specific number of parts. The simulation of this sequence of cycles is even more time demanding. Therefore, we evaluate the surrogate models in the prediction of the target variables for all the cycles in a batch. Since the objective is to effectively substitute the simulations, the surrogate model performs a sequence where the prediction of the next cycle is performed by taking as input the previous predictions.

For the reasons explained in Section 2.1, the validation sets have $t_{cool}=ctant$ along the whole batch, corresponding to real experimental cases where the transference of the sheet into the die is automatized and the forming time can be changed within the range of values given by the total cycle time. The validation sets consist of 14 batches for each one of the values of $t_{cool}$, which gives a total ratio of ∼1:2 with respect to the training sets.

Validation Scenario 4: Batches of $t_{cool}$ = 10 s, 15 s, and 20 s.

The validation set consists of batches of 50 cycles, where the cooling time is kept constant within the entire batch and it has values of $t_{cool}$ = 10 s, 15 s, and 20 s, the same ones as Training Set A. The forming time has a random value for each cycle, with the restriction of the cycle time $t_{cycle}=t_{form}+t_{cool}=\left[30,40\right]$ s. For each value of $t_{cool}$, we have 14 batches for validation.

**Figure 7:**
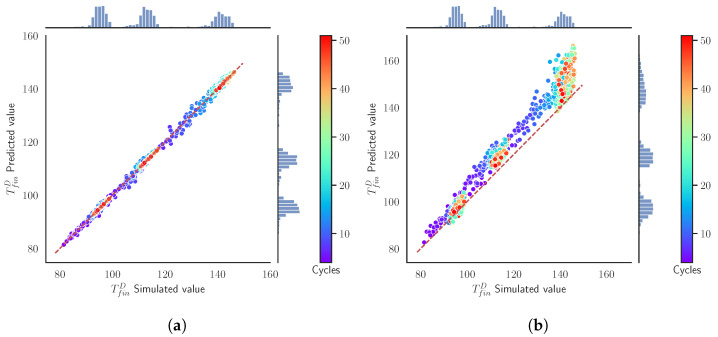
Validation Scenario 4: Predicted values as function of the simulated output values of the $T_{fin}^{D}$. The histograms represent the relative counts as function of the temperature and the color map indicates the cycle. (**a**) SModA and (**b**) SModB.

In Figure 7, we compare how both surrogate models predict the target variables $T_{fin}^{D}$, which defines the state of our system. The diagonal line acts as a reference of the perfect prediction. We can also observe the distribution of the simulated values and the predicted values in the histograms. Moreover, since we are evaluating batches of 50 cycles, the colors indicate the cycle of the prediction. Notice that the SModA is able to have a very good performance in this scenario. The reason is that it has been trained and finely tuned to those particular settings. The SModB predictions are shifted to higher values of $T_{fin}^{D}$ than our ground truth simulations, although the histograms are similar. The deviation from the diagonal becomes more evident in higher temperatures. In both Figure 7a and Figure 7b, the batches with different values of $t_{cool}$ can be identified, as higher values of $t_{cool}$ imply lower values of $T_{fin}^{D}$. Quantitatively, the MAE between the predictions and the simulated values for all the data of this validation scenario is presented Table 6, where the rest of target variables also have a very low value of MAE with the SModA.

**Figure 8:**
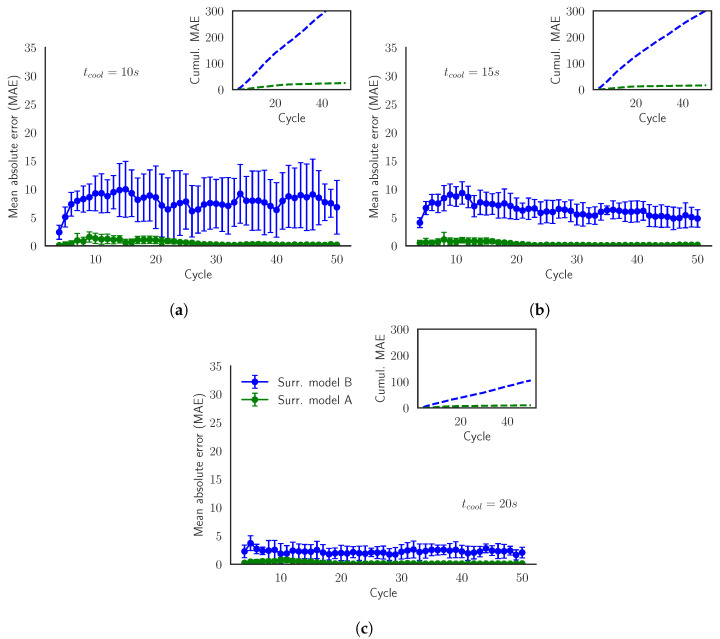
Validation Scenario 4: Mean absolute error (MAE) of the model prediction of $T_{fin}^{D}$ evaluated for batches with $t_{cool}=$ (**a**) 10 s, (**b**) 15 s, and (**c**) 20 s. The inner plot shows the evolution of the cumulative mean absolute error.

Figure 8 represents the MAE and the cumulative MAE of the predictions of $T_{fin}^{D}$ for each cycle within the batches in the validation set. We observe how the SModA has a nearly perfect prediction for the previously commented reasons. Furthermore, the error of SModB is accumulated in the first cycles and after that it remains constant or even decreases. Additionally, we confirm that this model works better for higher values of $t_{cool}$, i.e., for lower temperatures. The explanation can be found in the training sets. In Figure 3a (where the parameters are the same as in the current validation set), the stationary region of the curves of $t_{cool}$ = 10 s is not reached until cycle 15, in which the stationary region achieves temperatures around 140 °C. Figure 8 shows the important accumulation of error in the transient region for the SModB, and when $t_{cool}$ = 10 s, the transient region lasts more cycles. Besides, looking at Training Set B, the interval of temperatures around 140 °C in Figure 3b is not very populated. These are the main causes of the loss in the predictive power of the SModB for low values of $t_{cool}$. Summarizing, the transient region is the main source of error for the SModB, since the mean absolute error increases in the first cycles, while in the stationary region it is kept constant.

Validation Scenario 5: Batches of $t_{cool}=$ Intermediate.

In this case, the validation set is formed by batches of 50 cycles that have intermediate values of $t_{cool}$ = 11 s, 12 s, 13 s, 14 s, 16 s, 17 s, 18 s, and 19 s, and that are kept constant along the cycles. Therefore, the SModA is not trained with the same values of cooling time. The forming time has a random value for each cycle, but it is restricted by $t_{cycle}=t_{form}+t_{cool}=\left[30,40\right]$ s. For each value of $t_{cool}$, we have 14 batches.

**Figure 9:**
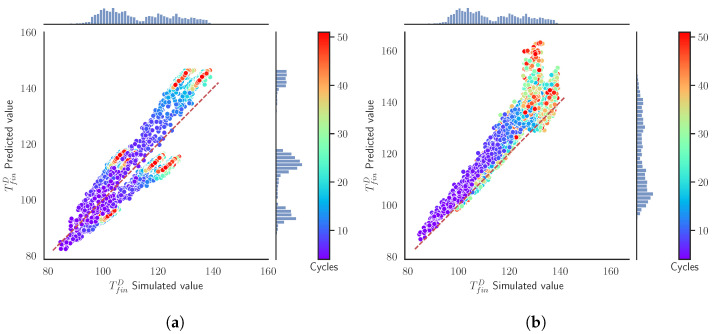
Validation Scenario 5: Predicted values as functions of the simulated output values of the $T_{fin}^{D}$. The histograms and the color map represent the relative counts as functions of the temperature. (**a**) SModA and (**b**) SModB.

In this scenario, Figure 9a evidences the lack of generalization of the SModA. We notice that the distribution of the predictions displayed in the vertical histogram has peaks in the same ranges of temperatures as the ones of Figure 7a. These ranges correspond to the stationary region of when $t_{cool}$ = 10 s, 15 s, and 20 s, implying that the SModA is not able to interpolate for intermediate values. In opposition, the predictions of the SModB present a similar temperature distribution to the simulated values. Additionally, comparing with Figure 7b of the previous validation scenario, we found an analogous behavior of the SModB in this case, as shown in the distribution of the points of Figure 9b. Focusing on Table 7, the comparison of the two surrogate models shows the lower values of MAE of the SModB with respect to the SModA for all the target variables for the intermediate values of $t_{cool}$. We notice that the values of MAE for the SModB are close to the ones in Table 6, which implies a comparable performance in both validation scenarios. Then, despite the small shift with respect to the diagonal line and the dispersion observed in high temperatures, the SModB is more convenient if we are seeking a model capable of generalizing within the defined range of the operational parameters.

**Figure 10:**
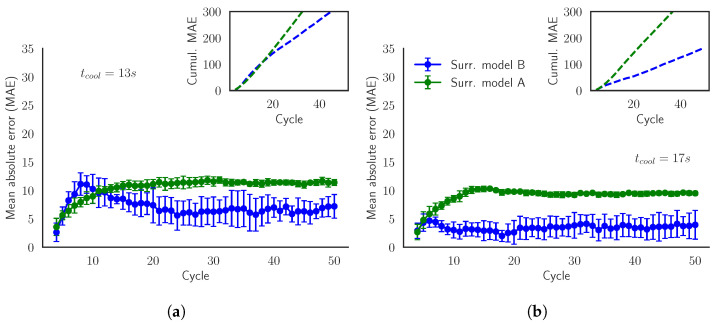
Validation Scenario 5: Mean absolute error (MAE) of the model predictions of $T_{fin}^{D}$ evaluated for the batches in the validation set with $t_{cool}=$ (**a**) 13 s and (**b**) 17 s. The inner plot shows the evolution of the cumulative mean absolute error.

The better performance of the SModB and its generalization potential are verified in Figure 10. Although the error increases in the first cycles, coinciding with the transient region, the SModB approaches the simulated values after that. On the contrary, the SModA error in the transient zone remains during the rest of the batch. As discussed, notice how the SModB works better for higher values of $t_{cool}$. After the evaluation of the model performance in the different scenarios, we choose the SModB over the SModA because it has shown to be a more general model. In spite of the remarkable generalization capability of the SModB, the model is far from being perfect, especially if we focus on Figure 10a. In the next section, we try to optimize the model performance and to reduce the computational time spent in the generation of the training set.

### 3.3. Model Optimization

Taking the SModB as the current baseline, in this section we aim to optimize this model, which has demonstrated a higher generalization capability than the SModA. However, the model has not been accurate enough in the prediction at high temperatures, i.e., low values of $t_{cool}$. Moreover, the current model is fed with the simulation of 270 batches of 50 cycles, which implies a lot of computational time. Therefore, there is a need to tune the model in order to reduce the computational time spent in the creation of the training set without a significant impact on the model accuracy. The performance of the model optimization criteria is evaluated in Validation Scenario 5, since it is the most adequate scenario for evaluating generalization in the batch prediction case.

#### 3.3.1. Number of Cycles in Training

**Figure 11:**
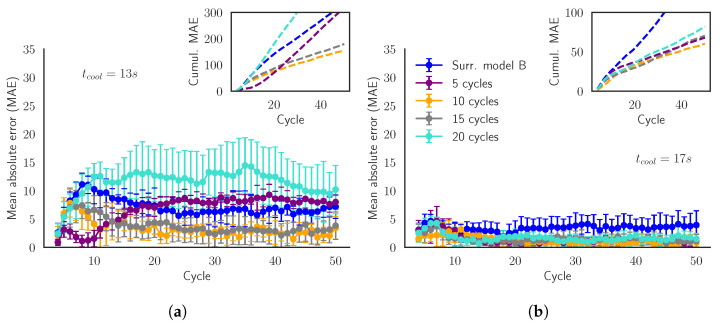
Validation Scenario 5: Mean absolute error (MAE) of the SModB and the models trained with less cycles. The predictions are evaluated for the batches in the validation set with $t_{cool}=$ (**a**) 13 s and (**b**) 17 s. The inner plot shows the evolution of the cumulative mean absolute error.

A reduction of the number of cycles of the batches of Training Set B implies training the surrogate model with less simulations. Table 8 shows that, in fact, this reduction not only decreases the time needed to create the training set but also it results in a better MAE. The surrogate model trained with only the first 10 cycles of the batches is the one that achieves the best MAE for all the target variables. This is verified in Figure 11, where for two arbitrary values of $t_{cool}$, the curves of MAE and the cumulative MAE of the model trained with batches of 10 cycles are displayed below the curves of the other models.

Looking at Figure 3b, we detect that once we reach the stationary region (around the 10th–15th cycle), the values of the temperatures are limited within an interval. Therefore, we have a biased training set with a lot of data inside the interval of temperatures of the stationary region. This bias leads to an overspecialization in the training phase, preventing good results in the transient state. Because errors at the transient state weigh a lot and are cumulative, the models trained with 10 or 15 cycles have better performances. In those training regimes, the model is fed with a better balance of both transient- and steady-state samples.

#### 3.3.2. Number of Simulated Batches in Training

**Figure 12:**
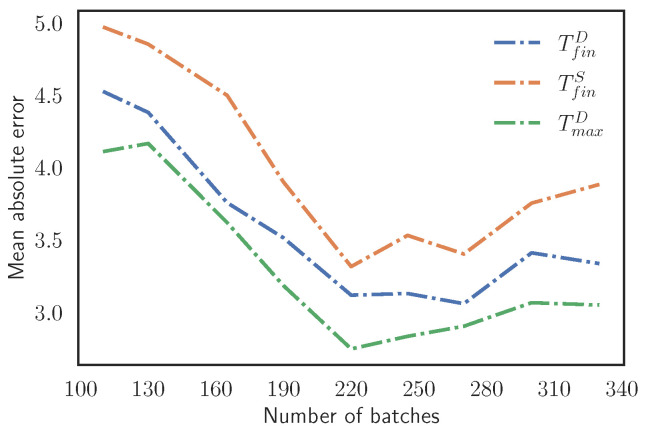
Evolution of the MAE for the three target variables evaluated in Validation Scenario 5 as we reduce the number of batches in the training set.

Once the use of the first 10 cycles to train the surrogate model is determined, we focus on the number of simulated batches that form the training set. By reducing the number of batches in the training set, less simulations are required, saving a lot of computational time for the creation of the training set. In Figure 12, we explore how the number of batches affects the model accuracy. Concretely, a threshold is found at around 220 batches in the training. As we decrease the number of batches down to this threshold, the MAE keeps increasing because the model does not have enough information to work properly.

**Figure 13:**
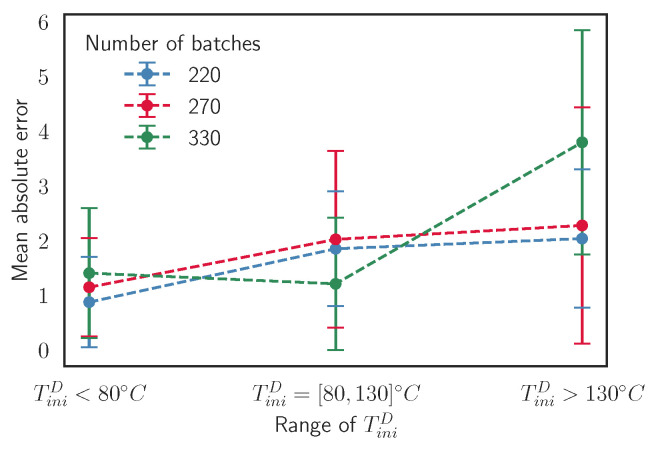
MAE for single predictions of $T_{fin}^{D}$ of samples of models trained with different numbers of batches. The sample parameters have the conditions determined in Validation Scenario 5 and they are classified depending on the input temperature of the die $T_{ini}^{D}$.

In addition, an increase of the simulated batches in the training set does not imply a better accuracy. The reason is that as we increase the number of data, the training set becomes more biased in the range of temperatures of the stationary region and the model is not able to capture the behavior well outside this range of temperatures. The effect of the bias is shown in Figure 13, where models trained with different numbers of batches perform a single-cycle prediction of different random cycles extracted from Validation Scenario 5. We define three intervals that classify the samples depending on the input $T_{ini}^{D}$: $T_{ini}^{D}<80$ °C, $T_{ini}^{D}=\left[80,130\right]$ °C, and $T_{ini}^{D}>130$ °C, and each interval has 20 samples. We notice how the models with 220 and 270 batches have similar behaviors, although the MAE is lower for the one trained with 220 batches. The model trained with more samples struggles in the intervals of low and high temperatures and it has a lower MAE in the intermediate interval than the other models. This verifies the existence of a bias in the training set that affects the predictions when the number of batches is too large. Then, as it happens in Section 3.3.1, the model suffers from overfitting, reducing its generalization capability.

#### 3.3.3. Final Surrogate Model

From the results of the previous Section 3.3.1 and Section 3.3.2, we have obtained an optimized surrogate model that is trained with less simulations and it has a better performance than the baseline SModB. This final surrogate model is trained with 220 batches of 10 cycles under the same conditions as Training Set B: the cooling time has a random value for each cycle within the range $t_{cool}=\left[0,40\right]$ and the cycle time also has a random value between $t_{cycle}=\left[30,40\right]$. The forming time is restricted by the condition of $t_{cycle}=t_{form}+t_{cool}$. The randomness of the input variables is the main cause for the generalization power of the final surrogate model.

**Figure 14:**
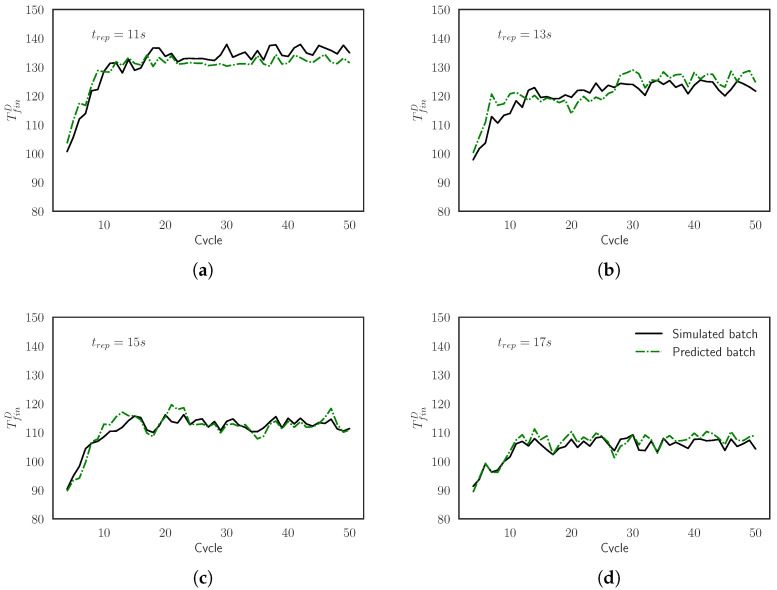
Comparison between the simulated curves and the predicted curves for the final surrogate model of $T_{fin}^{D}$ evaluated for batches with $t_{cool}=$ (**a**) 11 s, (**b**) 13 s, (**c**) 15 s and (**d**) 17 s.

In Figure 14, we have compared several curves of $T_{fin}^{D}$ from different simulated batches against the predictions of the model. The batches correspond to the case where $t_{cool}=ctant$, identified as a very interesting case to explore new possibilities in real industrial scenarios, as remarked in Section 2. We notice that the curves are very close and that the model is able to reproduce the simulation inside the whole range of $t_{cool}=\left[10,20\right]$.

Furthermore, the optimization of the baseline surrogate model to this final surrogate model has supposed a gain in the simulation time required to generate the training set. As mentioned, the CPU simulation time for a cycle is about 40 s. Initially, our baseline surrogate model was trained with 270 batches of 50 cycles, which are generated in approximately 150 h of CPU time. The training of the optimized model is performed with 270 of 10 cycles, decreasing the CPU time spent in simulation to ∼30 h. In addition, the 270 batches of 10 cycles have been reduced to 220 batches of 10 cycles for the training of the final surrogate model. Hence, we need less simulations to feed the model. This implies a reduction of almost ∼6 h in simulations, resulting in ∼24 h of CPU time spent in simulation. We have achieved a total reduction of ∼84% of the CPU time.

Despite the time spent in the simulations for the training, the major benefit of the final surrogate model is that it can generate a cycle or a batch four orders of magnitude faster than the simulations, as it is displayed in Table 9. It must be noticed that the geometry in this work is a metal sheet, and that a more complex geometry will enhance the need of surrogate modeling due to the increasing difficulty in FE simulations. Therefore, this creates a powerful tool to explore new operation scenarios, with a low computational and temporal cost. The aim of this exploration is to optimize the batch production with a direct effect in the improvement of the KPIs of the process. For instance, OEE could be improved by defining the optimal forming time for each cycle, reducing then the total cycle time and preventing the defects in the products, which leads to a better scrap rate. Moreover, this environment is very efficient to train promising data-driven technologies, such as digital twins or reinforcement learning agents, because it allows to reproduce a lot of batches under different parameter conditions. The remarkable soft real-time response that the surrogate model provides could be used by digital twins to predict the optimal operational configuration of the systems. In addition, reinforcement learning agents could be trained inside this efficient environment that mimics the real manufacturing system, and afterwards they could act in the real scenario, applying the learned policies and optimizing the process in the industrial plant. To sum up, the surrogate model opens the possibility to the introduction of self-autonomous systems in the press hardening process in industrial plants.

## 4. Conclusions

The wide range of applications of the press hardening process in the automotive industry and the extensive use of 22MnB5 in safety-related components highlight the importance of the process and the interest in ensuring the quality of the final products and a good manufacturing performance. In the actual paradigm of Industry 4.0, this work proposes a novel ML-based surrogate model to predict the most relevant results of the press hardening process of 22MnB5. The model has been validated in several scenarios and it is capable to provide a much faster response than simulation models. This enables the possibility to explore the parameter space and configurations in an efficient environment without the time limitations of the simulations. The current approach is innovative, since in previous works the applications of ML in the press hardening process have been based on the process monitoring and control at different points.

The model is trained with FE simulations and it is based on the XGBoost regression algorithm, which establishes relations between the input variables of the simulations with the most relevant process variables. The validation is performed in various feasible operational scenarios of a real plant, consisting in series of simulated batches of 50 cycles. In terms of the generalization of the surrogate model, it has been demonstrated that the training with batches with non-standard parameter conditions, which cover more regions of the parameter space, outperforms the training with batches with the standard parameter conditions.

Furthermore, the surrogate model has been optimized, decreasing the number of FE simulations required for its training. First, the number of cycles of the training batches has been reduced from 50 to 10 cycles. Remarkably, the surrogate model trained with batches with less cycles is able to reproduce larger batches more accurately in the validation scenarios. Next, the number of batches that feed the surrogate model has been decreased, and we have found a threshold of 220 batches before the accuracy starts to fall. This optimization has supposed an important decrease of 84% of the CPU time and has minimized the computational resources spent in the simulations needed to create the surrogate model.

Finally, the final optimized surrogate model is able to reproduce reasonably well the simulations inside the whole range of parameters of the real industrial plant. In fact, the models have the ability predict the target variables in the validation scenarios with a MAE of around 3 °C from the simulations, which is considered an acceptable error in an experimental context. The key advantage of the surrogate model is that it is four orders of magnitude faster than the simulations, triggering the exploration of new operation scenarios in an efficient environment. This opens the door to the setting of the optimal parameter values of the press hardening process, improving the KPIs of the batch production of the process. In addition, the surrogate model provides a soft real-time response, which is crucial for the development of tools such as digital twins or reinforcement learning agents. These promising technologies demand a fast and efficient environment that act as a representation of a real scenario to be trained. Surrogate modeling will also be required in the introduction of other geometries in the press hardening process due to the increasing complexity. To recap, the surrogate model methodology proposed in the work enables the self-autonomous system’s presence in the press hardening process industrial plants, with the possibility to be expanded to other manufacturing conditions or processes.

## Figures and Tables

**Table 1:** Typical chemical composition in % of 22MnB5 sheet steel and 1.2344 tool steel.

Element	C	Si	Mn	Cr	B	Mo	V
22MnB5	0.20–0.25	0.15–0.35	1.1–1.4	0.15–0.30	0.002–0.004		
1.2344	0.39	1.00		5.40		1.35	1.00

**Table 2:** MAE and standard deviation (SD) results from the five-fold CV for the different target variables and the four candidate regression algorithms: K-nearest neighbours (KNN), gradient boosting (XGBoost), support vector machine (SVR), and random forest.

Surrogate Model	Target Variable	KNN	XGBoost	SVR	RF
	$T_{fin}^{D}$	0.261(0.009)	0.161(0.006)	1.002(0.010)	0.184(0.006)
A	$T_{fin}^{S}$	0.797(0.012)	0.157(0.004)	2.287(0.074)	0.203(0.006)
	$T_{max}^{D}$	0.205(0.005392)	0.045(0.002)	0.127(0.026)	0.055(0.002)
	$T_{fin}^{D}$	2.075(0.013)	1.674(0.021)	2.615(0.026)	1.853(0.015)
B	$T_{fin}^{S}$	8.703(0.421)	1.433(0.035)	34.993(1.148)	1.769(0.022)
	$T_{max}^{D}$	1.796(0.064)	0.768(0.023)	5.141839(0.202)	0.871(0.029)

**Table 3:** MAE and SD results for the different target variables and the two surrogate models in the next cycle prediction for Validation Scenario 1.

Target Variable	SModA	SModB
$T_{fin}^{D}$	0.172(0.200)	1.781(1.475)
$T_{fin}^{S}$	0.193(0.246)	1.893(1.417)
$T_{max}^{D}$	0.054(0.069)	1.055(0.778)

**Table 4:** MAE and SD results for the different target variables and the two surrogate models in the single-cycle prediction for Validation Scenario 2.

Target Variable	SModA	SModB
$T_{fin}^{D}$	3.190(1.607)	1.939(1.559)
$T_{fin}^{S}$	1.179(0.912)	2.308(1.521)
$T_{max}^{D}$	0.506(0.376)	1.160(0.728)

**Table 5:** MAE and SD results for the different target variables and the two surrogate models in the single-cycle prediction for Validation Scenario 3.

Target Variable	SModA	SModB
$T_{fin}^{D}$	12.536(10.429)	2.153(3.274)
$T_{fin}^{S}$	60.157(117.197)	2.665(13.875)
$T_{max}^{D}$	8.023(17.931)	2.078(9.802)

**Table 6:** MAE and SD results for the different target variables and the two surrogate models in the batch prediction for the Validation Scenario 4 data.

Target Variable	SModA	SModB
$T_{fin}^{D}$	0.359(0.492)	5.484(3.886)
$T_{fin}^{S}$	0.391(0.518)	6.335(4.470)
$T_{max}^{D}$	0.293(0.412)	5.362(3.586)

**Table 7:** MAE and SD results for the different target variables and the two surrogate models in the batch prediction for the Validation Scenario 5 data.

Target Variable	SModA	SModB
$T_{fin}^{D}$	7.598(3.515)	5.605(4.760)
$T_{fin}^{S}$	8.434(2.961)	6.796(5.051)
$T_{max}^{D}$	7.044(2.909)	5.554(4.309)

**Table 8:** MAE and SD results of the model predictions of $T_{fin}^{D}$ for the different target variables in the batch prediction for the Validation Scenario 5 data as we decrease the number of cycles of the batches of Training Set B.

Target Variable	Surr. Model B (50 Cycles)	20 Cycles	15 Cycles	10 Cycles	5 Cycles
$T_{fin}^{D}$	5.605(4.760)	5.395(5.377)	3.398(2.983)	3.058(2.291)	5.315770(4.324)
$T_{fin}^{S}$	6.796(5.051)	6.045(5.962)	4.360(3.592)	3.402(2.457)	4.686(3.781)
$T_{max}^{D}$	5.554(4.309)	4.855(5.084)	3.305(2.894)	2.903(2.013)	3.983(3.207)

**Table 9:** Comparison between the simulation times and the final surrogate model times in cycle and batch generation.

Method	Cycle Time	Batch Time
Simulation	∼40 s	∼2000 s
Final Surrogate Model	∼3 × 10^−3^ s	∼1.5 × 10^−1^ s

**Figure A1:**
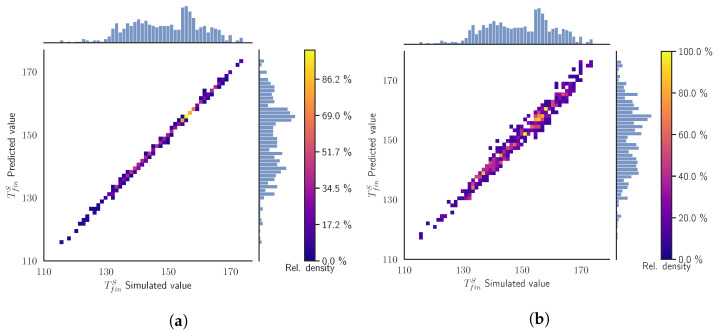
Validation scenario 1: Predicted values as function of the simulated output values of the $T_{fin}^{S}$. The histograms and the color map represent the relative counts as function of the temperature. (**a**) SModA and (**b**) SModB.

**Figure A2:**
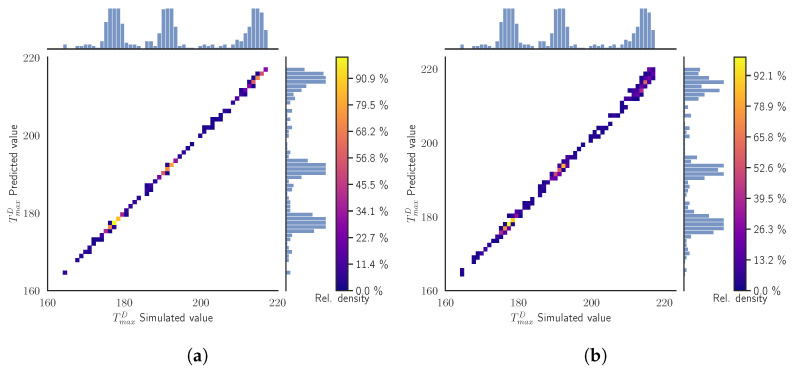
Validation scenario 1: Predicted values as function of the simulated output values of the $T_{max}^{D}$. The histograms and the color map represent the relative counts as function of the temperature. (**a**) SModA and (**b**) SModB.

**Figure A3:**
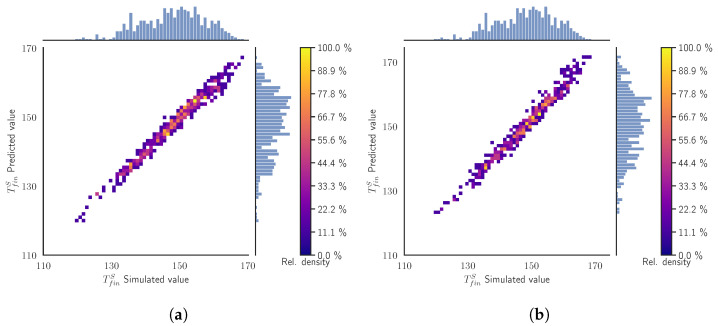
Validation scenario 2: Predicted values as function of the simulated output values of the $T_{fin}^{S}$ under. The histograms and the color map represent the relative counts as function of the temperature. (**a**) SModA and (**b**) SModB.

**Figure A4:**
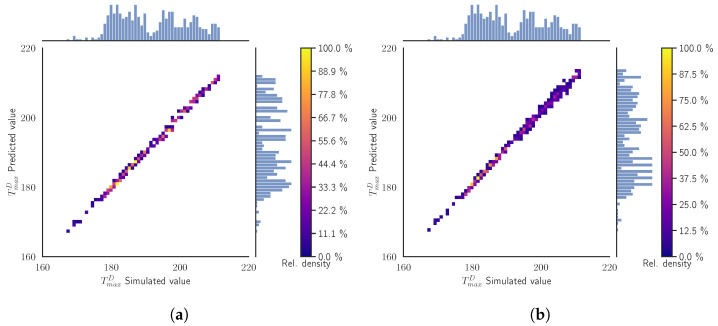
Validation scenario 2: Predicted values as function of the simulated output values of the $T_{max}^{D}$ under. The histograms and the color map represent the relative counts as function of the temperature. (**a**) SModA and (**b**) SModB.

**Figure A5:**
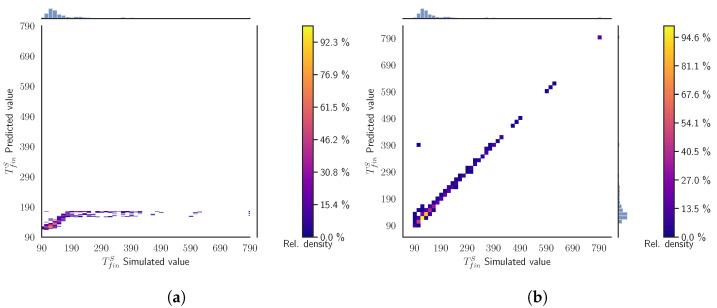
Validation scenario 3: Predicted values as function of the simulated output values of the $T_{fin}^{S}$. The histograms and the color map represent the relative counts as function of the temperature. (**a**) SModA and (**b**) SModB.

**Figure A6:**
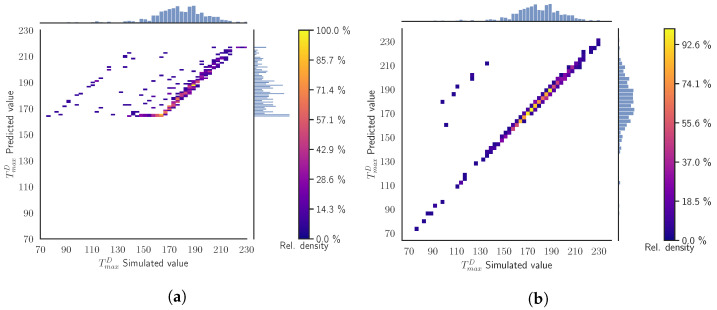
Validation scenario 3: Predicted values as function of the simulated output values of the $T_{max}^{D}$. The histograms and the color map represent the relative counts as function of the temperature. (**a**) SModA and (**b**) SModB.

**Figure A7:**
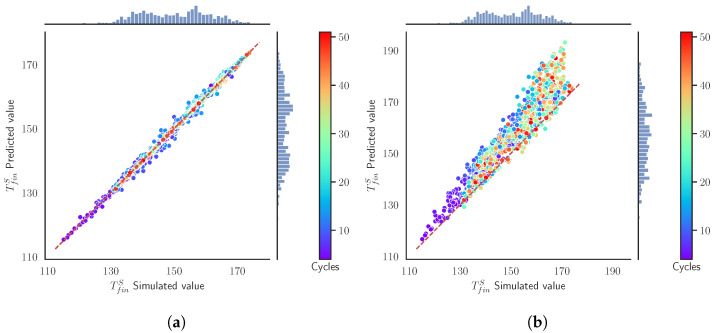
Validation scenario 4: Predicted values as function of the simulated output values of the $T_{fin}^{S}$. The histograms represent the relative counts as function of the temperature and the color map indicates the cycle. (**a**) SModA and (**b**) SModB.

**Figure A8:**
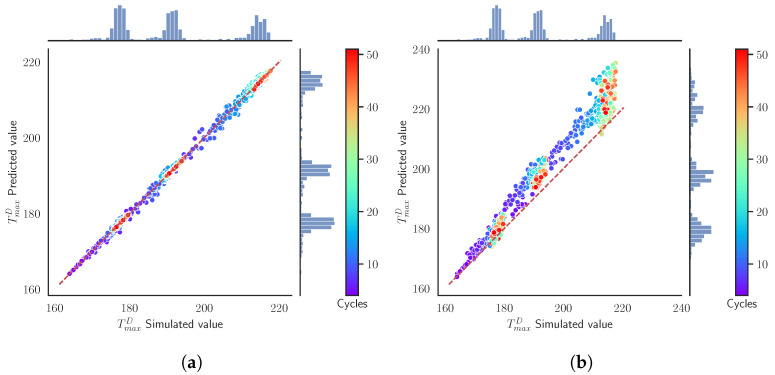
Validation scenario 4: Predicted values as function of the simulated output values of the $T_{max}^{D}$. The histograms represent the relative counts as function of the temperature and the color map indicates the cycle. (**a**) SModA and (**b**) SModB.

**Figure A9:**
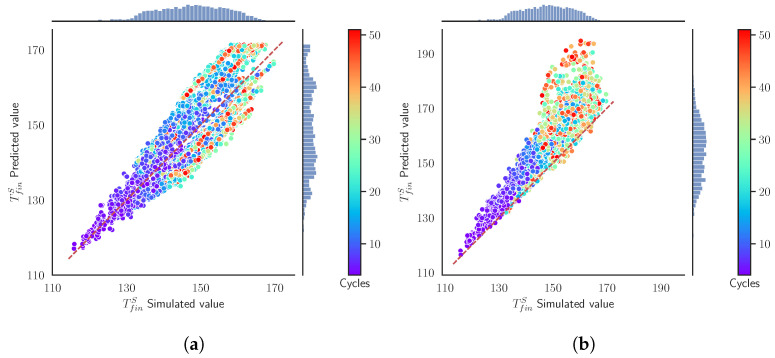
Validation scenario 5: Predicted values as function of the simulated output values of the $T_{fin}^{S}$. The histograms and the color map represent the relative counts as function of the temperature. (**a**) SModA and (**b**) SModB.

**Figure A10:**
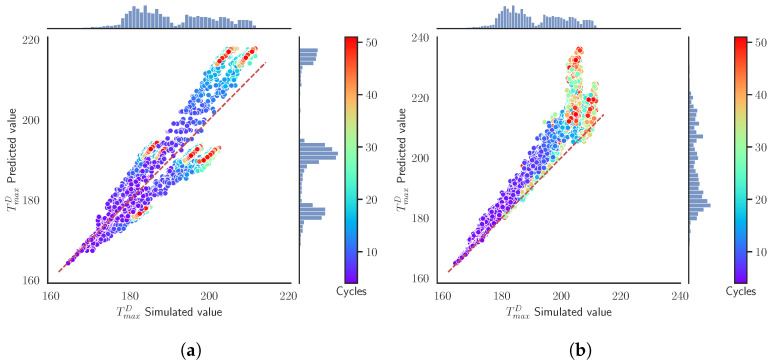
Validation scenario 5: Predicted values as function of the simulated output values of the $T_{max}^{D}$. The histograms and the color map represent the relative counts as function of the temperature. (**a**) SModA and (**b**) SModB.

**Figure A11:**
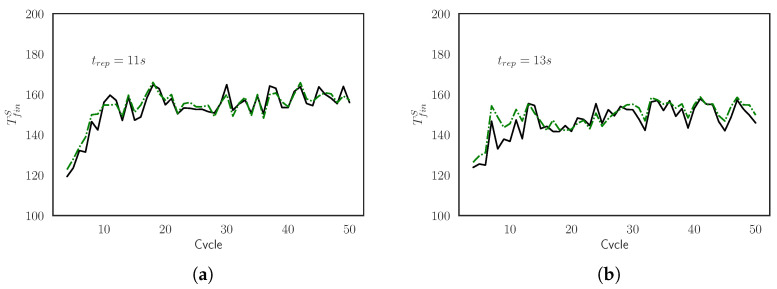

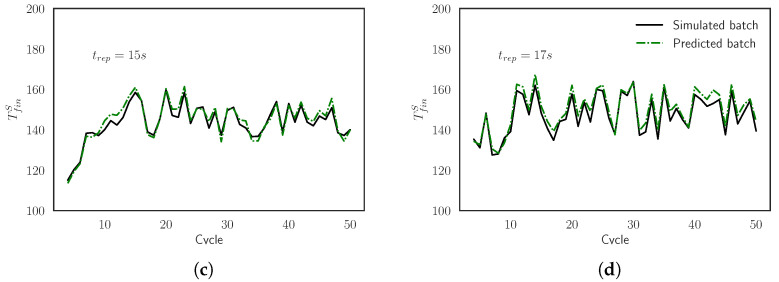
Comparison between the simulated curves and the predicted curves for the final surrogate model of $T_{fin}^{S}$ evaluated for batches with $t_{cool}=$ (**a**) 11 s, (**b**) 13 s, (**c**) 15 s, and (**d**) 17 s.

**Figure A12:**
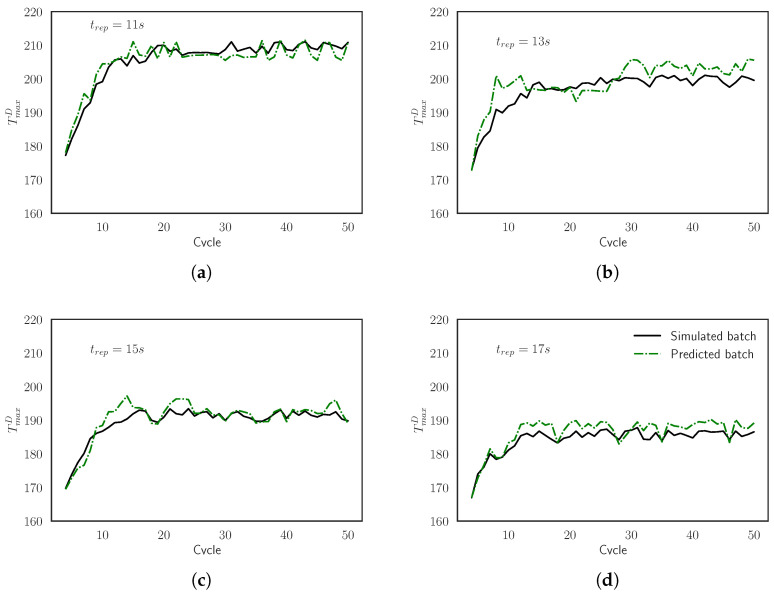
Comparison between the simulated curves and the predicted curves for the final surrogate model of $T_{max}^{D}$ evaluated for batches with $t_{cool}=$ (**a**) 11 s, (**b**) 13 s, (**c**) 15 s, and (**d**) 17 s.

## Data Availability

The data presented in this study are available upon reasonable request from the corresponding author.

## Abbreviations

The following abbreviations are used in this manuscript:

UHSS	Ultra-high-strength steels
ML	Machine learning
AI	Artificial intelligence
GANs	Generative adversarial networks
ANNs	Artificial neural networks
KPI	Key performance indicators
OEE	Overall equipment effectiveness
FE	Finite element
FEM	Finite element modeling
SModA	Surrogate model A
SModB	Surrogate model B
KNN	K-nearest-neighbors
XGBoost	Extreme gradient boosting
SVR	Support vector regressor
RF	Random forest
MAE	Mean absolute error
SD	Standard deviation
CV	Cross-validation

## Appendix A

In this section, the figures of the target variables of the press hardening process $T_{fin}^{S}$ and $T_{max}^{D}$ are presented.
